# Iron oxide nanoparticles as multimodal imaging tools

**DOI:** 10.1039/c9ra08612a

**Published:** 2019-12-06

**Authors:** Edouard Alphandéry

**Affiliations:** Paris Sorbonne Université, Muséum National d'Histoire Naturelle, UMR CNRS7590, IRD, Institut de Minéralogie, de Physique des Matériaux et deCosmochimie, IMPMC 75005 Paris France; Nanobacterie SARL 36 Boulevard Flandrin 75116 Paris France; Institute of Anatomy, UZH University of Zurich, Instiute of Anatomy Winterthurerstrasse 190 CH-8057 Zurich Switzerland edouardalphandery@hotmail.com +4133632697020

## Abstract

In medicine, obtaining simply a resolute and accurate image of an organ of interest is a real challenge. To achieve this, it has recently been proposed to use combined methods in which standard imaging (MRI, PAI, CT, PET/SPEC, USI, OI) is carried out in the presence of iron oxide nanoparticles, thus making it possible to image certain tissues/cells through the specific targeting of these nanoparticles, hence resulting in improved imaging contrast and resolution. Here, the advantages and drawbacks of these combined methods are presented as well as some of their recent medical applications.

## Abstract 2

In medicine, obtaining simply a resolute and accurate image of an organ of interest is a real challenge.

## Graphical abstract

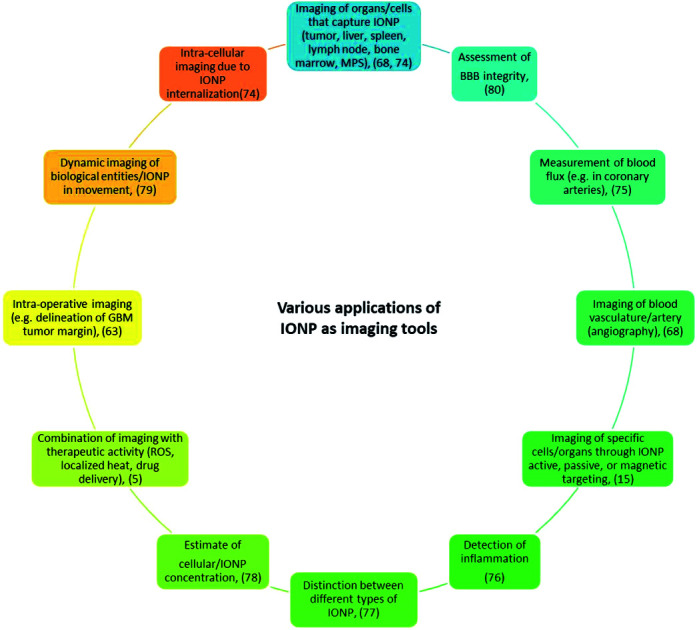

## Introduction

**Fig. 1:**
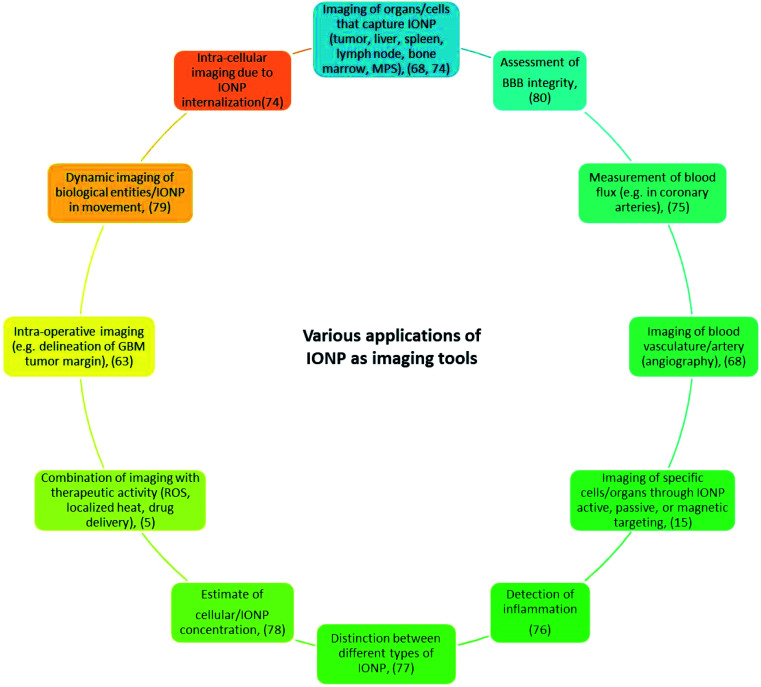
Various applications of IONP as imaging tools.

Iron oxide nanoparticles (IONP) can be used to image organs/cells, which capture or accumulate them, such as the liver,^1^ spleen,^2^ lymph nodes,^3,4^ bone marrow, and those of the mononuclear phagocytic system, whether these organs/cells are tumorigenic or not.^5,6^ Other examples of IONP imaging applications include the detection of apoptosis,^7^ inflammation,^8^ angiography,^9^ ruptured atherosclerotic plaque,^10^ multiple sclerosis,^5^ integrity of the blood–brain barrier,^11^ and vasculature, *e.g.* coronary arteries^12^ (Fig. 1). The development of IONP for these applications stems from their advantages compared with non-nanoparticle based systems such as: (i) their longer residence/circulation time,^13^ (ii) their faculty to act as a contrast agent for several imaging methods simultaneously,^14^ (iii) their ability to specifically target an organ/tissue of interest *via* passive, active or magnetic targeting with an efficacy that varies depending on studies and leads to a percentage of IONPs that target the tumor relative to the quantity of injected IONP that is between 4 × 10^−4^% and 7%,^15^ and (iv) their use as ‘theranostic’ probes, where IONP therapeutic activity comes from localized ROS or heat production, exposure of IONP to various excitation sources, or conjugation of drugs to IONP.^16^ Furthermore, since IONP are already used on humans for therapeutic applications either to treat iron anemia disease or to carry out magnetic hyperthermia treatment of cancer,^15^ one could easily foresee their clinical use for imaging applications.

Here, I review the use of IONP as imaging tools in various imaging methods, *i.e.* magnetic resonance imaging (MRI), magnetic particle imaging (MPI), photo-acoustic imaging (PAI), computing tomography (CT), positron emission tomography (PET)/single photon emission computed tomography (SPECT), ultrasound imaging (USI), and optical imaging (OI). IONP appeal comes from the fact that they enable:

• Adjustment of IONP *T*_1_/*T*_2_ contrasting strength by tuning the properties of these nanoparticles (size, charge, assembly, surface) for MRI;^17^

• Use of a device generating a magnetic field that can both image and heat IONP through magnetic hyperthermia for MPI;^18^

• Improved imaging resolution using common diagnostic devices for most imaging methods;^19^

• Localized functional imaging for PET/SPECT;^20^

• A broad spectrum of different types of detections, *e.g.* drug release and intracellular imaging, for optical imaging.^21^

The review presented here is broader in scope than previous ones since it covers more imaging techniques and is not restricted to IONP synthesized by a specific method.^22–25^

**Fig. 2:**
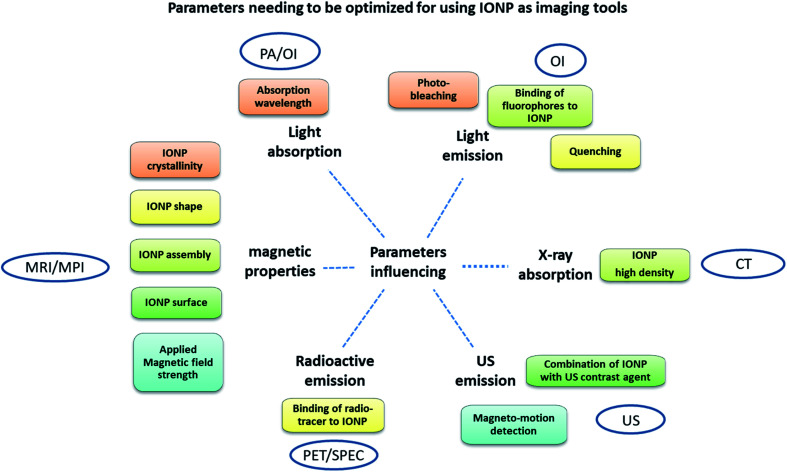
Parameters that need to be optimized for using IONP as imaging tools in PA, OI, CT, USI, PET/SPEC, MRI, MPI.

INOP advantages/drawbacks and parameters influencing IONP their imaging power in these various imaging methods are summarized in Table 1 and Fig. 2.

**Table 1:** Advantages and drawbacks of the various imaging techniques (PA, OI, CT, USI, PET/SPEC, MRI, MPI) used in combination with IONP

Imaging methods	Advantages	Drawbacks
MRI	Widely used (available clinically)	Negative contrast
	High penetration depth	Artifacts preventing detailed anatomical imaging
	Rapid signal acquisition	No direct measurement of concentration
	Functional information using fMRI	Not so good resolution (25–100 μM)
	Large area covered with one scan (>2500 cm^2^)	
MPI	No penetration depth limit	Slow signal acquisition
	Direct measurement of concentration	No widely used (not available clinically)
	No radiation	No functional information
	Good resolution (∼1 mm)	
	Can combine imaging/therapeutic activity (MPH)	
	Large area covered with one scan (can be adjusted by varying coil size)	
PAI	No radiation	Low penetration depth (∼5 cm)
	Rapid signal acquisition	Not widely used (only prototypes available in clinic)
	Good resolution (∼0.5 nm)	No direct measurement of IONP concentration
	Large area covered with one scan (∼15 cm^2^)	
	Functional information	
CT	Large area covered with one scan (>2500 cm^2^, similar to MRI)	
	Rapid signal acquisition	Not so good resolution (5–200 μm)
	No penetration depth limit	Radiation
	Widely used (available clinically)	No functional information
	Large area covered with one scan?	No direct measurement of low IONP concentration
		Limited soft tissue resolution
PET/SPECT	Widely used (available clinically)	Radiation
	Simultaneous anatomical/functional imaging	Not so good good resolution (2-10 mm)
	Can combine imaging/therapeutic activity	Not straightforward to bind a radiotreacer to IONP and maintain the complex IONP-radiotrace active *in vivo* without its destruction
	No penetration depth limit	Not straightforward to acquire signal
	Can cover the whole body	
	No radiation	
	Direct measurement of concentration in some cases	
Sonography	Widely used (available clinically)	No direct detection (use of magneto-motive mechanism or additional contrast agent such as micro-bubble)
	No radiation	Not so good resolution (50–500 μM)
	Rapid signal acquisition	Limited penetration depth (∼5–20 cm)
	Large area covered with one scan (∼200 cm^2^)	No functional information
		No direct measurement of IONP concentration
Optical method	No radiation	Limited penetration depth (∼1–5 cm)
	Area covered depends on diameter/number of optical fibers used	Not so good resolution (1–5 mm)
	Can combine therapeutic/imaging activity	Not widely used clinically
	Functional information in some cases	Not straightforward to bind a fluorophore to IONP and maintain the complex IONP-fluorophore luminescent in vivo
	Measurement of IONP concentration in some cases	Not straightforward to acquire signal
All techniques	Can be combined with eachother	One technique does not gather all advantages
		IONP can be eliminated in blood (capture by macrophages/opsonization)

### Magnetic resonance imaging (MRI)

Since human body is mainly composed of water, specific water properties could be measured to image all parts of the organism. MRI was developed to measure proton relaxation times of water molecules following a two-steps excitation process in which a static magnetic field (*B*_0_) first produces longitudinal magnetization, *i.e.* alignment of proton nuclear spins parallel to *B*_0_, and then a radiofrequency pulse yields transverse magnetization, *i.e.* alignment of proton nuclear spins perpendicular to *B*_0_. Upon removal of the radiofrequency pulse, the proton nuclear spins relax longitudinally and transversely with relaxation times of *T*_1_ and *T*_2_, respectively. IONP can be used as contrast agents to improve the quality of MRI images by decreasing the values of *T*_1_ and/or *T*_2_. IONP contrast agents (CA) can be divided between *T*_1_ and *T*_2_ CA that lead to bright and dark MRI images associated with the recovery of the longitudinal magnetization (positive contrast, *T*_1_) or loss of transverse magnetization (negative contrast, *T*_2_). The relaxivity of an assembly of IONP and water molecules (*R*_*i*_) can be expressed as a function of the relaxivities of isolated water protons (*R*_water_) and IONP magnetic moment (*R*_IONP_), using the relation: *R*_*i*_ = 1/*T*_*i*_ = *R*_water_ + *C*_IONP_*R*_IONP_ (*i* = 1 or 2). This relation indicates how *R*_*i*_ can be maximized by using large values of *R*_IONP_ and *C*_IONP_. Furthermore, on the one hand, the Solomon–Bloemberger–Morgan theory indicates that *R*_1_ can be maximized by increasing IONP proton molecular thumbling time and decreasing proton residence lifetime. On the other hand, the outer-sphere diffusion model suggests that the effect of IONP on *T*_1_ or *T*_2_ relaxivities essentially depends on the stability or strength of IONP magnetic moment. According to this model, IONP with less stable magnetic moments and smaller sizes essentially increase *T*_1_ relaxivities while IONP with more stable magnetic moments and larger sizes increase *T*_2_ relaxivities. However, these theories are simplified. They don't take into account the whole stet of parameters that can influence the values of *T*_1_ and *T*_2_ relaxivities, which include:^26,27^

• Size of IONP, with small IONP acting as a positive contrast agent, *e.g. r*_1_ increases from 3–30 mM^−1^ s^−1^ for IONP of ∼20–65 nm to 66 mM^−1^ s^−1^ for IONP of ∼5 nm,^28^ and large IONP behaving as negative contrast agents, *e.g. r*_2_ increases from 35–130 mM^−1^ s^−1^ for IONP of 4–5 nm to 218–385 mM^−1^ s^−1^ for IONP of 12–14 nm,^28^

• Shape of IONP, which affects the stability of IONP magnetic moment and magnetization, leading “theoretically” to well-distributed magnetization for ellipsoids and to localization of magnetization in specific locations of IONP for other geometries, *e.g.* in the corners of IONP cubes, a parameter that seems to influence IONP contrasting power if water molecules are unable to reside where IONP magnetization is located.

• Crystallinity of IONP, which determines whether IONP affects *T*_1_ and/or *T*_2_ through: (i) IONP crystal phase, *e.g.* spinel/inverse spinel crystal phases of Fe_2_O_3_/Fe_3_O_4_ with ferrimagnetic behaviors enhance *T*_2_ contrast while IONP with antiferromagnetic behaviors, low magnetic moments, and small sizes such as α-FeOOH mainly impact *T*_1_ contrast, (ii) the presence of doping material in the crystal, *e.g.* doping XFe_2_O_4_ with X = Mn^2+^, Fe^2+^, Co^2+^, or Ni^2+^ leads to the largest magnetic moment for X = Mn^2+^ and a very significant effect on *T*_2_ with *r*_2_ = 358 mM^−1^ s^−1^ (1.5 T), and most interestingly (iii) the formation of mixed crystalline structures such as Gd_*x*_Fe_*y*_O_*z*_, possibly enabling dual-mode *T*_1_–*T*_2_ contrast agents.

• Surface of IONP, which can lead to an enhanced IONP contrasting ability when: (i) surface defects are minimized, (ii) the number of metallic atoms at IONP surface that are close to water molecules is maximized, (iii) a specific binding material covers the surface, (iv) oxygen atoms occupy surface vacancies in a well-adjusted manner, (iv) coating material and coating thickness are chosen to optimize the interactions between IONP magnetic moments and protons of water molecules.

• Assembly of IONP, which can change IONP contrasting power by modifying the diffusion properties of either IONP or water molecules surrounding IONP or both, *e.g.* a decrease in diffusion of water molecules surrounding IONP assemblies was associated with an increase in *T*_2_.

While some of these parameters favor one type of contrast to the detriment of the other one, *e.g.* a large nanoparticle size could enhance negative contrast and decrease positive contrast, other ones such as a mixed nanoparticle structure containing both metallic (Iron) and Gd atoms can promote simultaneously *T*_1_ and *T*_2_ contrasting ability.

In addition, since IONP contrasting ability depends on IONP interaction with the excitation sources, *i.e.* a combination of magnetic field and radiofrequency, the parameters of the excitation sources, in particular the strength or homogeneity/inhomogeneity of the magnetic field applied or the temperature of measurement, are other important parameters that influence the values of *T*_1_ and *T*_2_.

IONP, commercialized as negative or positive contrast agents, are listed in Table 2. They have been used for imaging: (i) liver/spleen using Ferumoxides/AMI-24/AMI-25/Endorem/Feridex or Ferucarbotran/SHU555A/Resovist/Cliavist,^29^ (ii) lymph node/bone marrow imaging using Ferumoxtran-10/AMI-227/Sinerem/Combidex or Ferucarbotran/SHU555C/Supravist,^30^ (iii) angiography using Feruglose/NC100250/Clariscan or Ferumoxytol/AMI-7228/Feraheme (231), (iv) delineation of the bowel from adjacent organs and tissues, using AMI-121/Lumirem/Gastromark,^32^

**Table 2:** Properties of IONP as contrast agents in MRI, such as IONP blood half-life, values of IONP relaxivities *r*_1_ and *r*_2_, recommended IONP clinical dose expressed in quantity of iron in IONP per Kg of patients, administration route, *i.e.* intravenous (iv), intra-gastric (IG), as well as IONP applications for imaging various parts of the organism with MRI

Properties as MRI contrast agents of commercialized iron oxide nanoparticles							
Name/reference	Composition	Blood half life in patients	*r*_1_ (mmol^−1^ s^−1^)	*r*_2_ (mmol^−1^ s^−1^)	Type of contrast	Clinical dose	Application/administration
Ferumoxides (AMI-24 AMI-25), endorem, feridex	SPION (120–180 nm) + dextran coating (ref. 66)	10 min (ref. 65)	24 (ref. 68 and 73)	100–160 (ref. 72 and 73)	Negative	30 μmol Fe kg^−1^ (ref. 66)	Liver/spleen I (ref. 69)
Ferucarbotran SHU555A, Resovist/Cliavist	SPION (60–80 nm) + carboxy-dextran coating (ref. 66)	12 min (ref. 65)	25 (ref. 68 and 73)	164–177 (ref. 72 and 73)	Negative	8–12 μmol Fe kg^−1^ (ref. 66)	Liver/spleen IV (ref. 68 and 69)
Ferumoxtran-10 AMI-227, Sinerem Combidex	USPIO (30 nm) + dextran coating (ref. 66)	24–30 h (ref. 65)	22 (ref. 68 and 73)	44–85 (ref. 72 and 73)	Positive or negative	45 μmol Fe kg^−1^ (ref. 66)	Lymph node bone marrow IV (ref. 68 and 69)
Ferucarbotran SHU555C, Supravist	USPIO (20–25 nm) + carboxy-dextran coating (ref. 66)	6–8 h (ref. 65)	7 (ref. 68)	57 (ref. 68)	Positive	40 μmol Fe kg^−1^ (ref. 66)	Perfusion lymph node bone marrow IV (ref. 68 and 69)
Feruglose NC100250, Clariscan	USPIO (10–20 nm) + carbohydrate-polyethylene glycol (ref. 66)	2 h (ref. 65)	20 (ref. 68)	35 (ref. 68)	Positive	36 μmol Fe kg^−1^ (ref. 66)	Perfusion angiography IV (ref. 68 and 69)
Ferumoxytol AMI-7228, Feraheme	USPIO (3 nm) + carboxy-methyldextran (ref. 67)	14 h (ref. 65)	15 (ref. 71)	89 (ref. 71)	Positive	50–400 mg per patient (ref. 71)	Angiography IV (ref. 68 and 70)
AMI-121 Lumirem and Gastromark	SPION (300 nm) + silica coating (ref. 74)	NA	3 (ref. 68)	72 (ref. 68)	Negative	105 mg per patient (ref. 74)	GI oral (ref. 74, 69 and 31)

The commercialization of most IONP mentioned in Table 2 has stopped for the following reasons.^33^ First, IONP are often eliminated in blood where they are captured by macrophages, making the targeting of specific organs by IONP a difficult task. Second, IONP can result in the formation of artifacts/black holes, which can prevent the realization of a detailed image of anatomical/tissular structures. Third, a quantity/concentration of IONP can't be deduced from an MRI image since IONP only indirectly affect an MRI image. Fourth, most IONP are *T*_2_ contrast agents, which are not favored by clinicians due to their darkening contrasting effect on an MRI image.

### MPI (magnetic particle imaging)

Magnetic particle imaging (MPI)^34,35^ is a new appealing imaging technique, which relies on the application of two types magnetic fields. A first magnetic field gradient results in the formation of a first region where the strength of the magnetic field is sufficiently high to saturate IONP magnetic moments and align them parallel to the applied magnetic field, and a second region, also called field free point (FFP), where the magnetic field strength is zero, and IONP magnetic moments are randomly oriented. When IONP located in the FFP are exposed to a second oscillating magnetic field of strength 10–100 mT and frequency 10–100 KHz,^36^ their magnetization oscillates with time, leading to an electromagnetic signal, which is detected and further converted into an MPI image. As for MRI, the quality of MPI imaging depends on IONP properties, such as IONP size, leading to an increase in spatial resolution by a factor 4 using IONP of 20 nm compared with Resovist of 45–60 nm. Several applications of IONP in MPI have been suggested such as:

• Cell concentration measurement, *i.e.* it is possible to obtain a relation between the MPI signal and the cellular concentration,^37^ with a high resolution, down to ∼250 cells and 7.8 ng of Fe. It however necessitates knowing on the one hand the quantity of IONP internalized in each cell and on the other hand how IONP internalization affects MPI signal. Whereas the quantity of IONP nanoparticles internalized in each cell can easily be determined *in vitro*, it seems more difficult to evaluate it *in vivo* without extracting a tissue sample from the organism for analysis, restricting the use of this method. In addition, whereas the MPI signal of some IONP is relatively independent of their environment and of the fact that they are internalized or not, due to a minor Brownian contribution to their MPI signal, other IONP display a MPI signal that strongly depends on IONP environment,^38^ making the judicious choice of IONP crucial for accurate MPI measurement.

• Imaging of several different types of IONP, since IONP with different sizes yield different MPI signals. This can enable the detection of various biological entities if each IONP of different size targets a different entity.^39^

• Measurement of blood flux, which can on the one hand enable evaluating the displacement speed of IONP in blood by MPI and on the other hand certain specific blood trajectory such as that resulting from bleeding, *i.e.* IONP leakage outside of blood vessels.^40^

• Imaging of some organs or tissues (tumors), which are either targeted by IONP following different targeting methods, *i.e.* passive through the EPR effect, magnetic *via* the application of a magnetic field gradient, or active using an antibody bound to IONP that targets a specific cell receptor, or directly injected with IONP, yielding imaging of organ, *e.g.* lung^41^ or brain,^42^ or vasculature,^37^ imaging without facing the same hurdle as in MRI in which IONP can leads to a hypo-intense signal that is similar to that of air inside lung.

• MPI combined with MHT, which can be foreseen since the properties of the applied oscillating magnetic field (strength and frequency) are similar in MPI and MHT (magnetic hyperthermia), possibly enabling combining imaging and therapeutic methods with IONP.^18^

MPI present a series of advantages such as: (i) a high penetration depth due to a theoretical unlimited propagation of the magnetic field, (ii) a direct measurement of IONP concentration, (iii) a positive contrast, (iv) an operating mechanism that does not rely on the use of toxic radiations, (v) a signal that should not be strongly affected by background tissue, (vi) a signal that can be acquired quickly in a spatially selective manner, (vii) a high resolution (sub-mm). However, MPI also suffers from a series of drawbacks such as: (i) the absence of anatomic imaging (MPI only images IONP), (ii) possible formation of eddy currents that can yield overheating if the strength/frequency of the oscillating magnetic field is too high, (iii) the necessity to use IONP whose magnetic moment couples with the external oscillating magnetic field without depending on IONP environment, (iv) the absence of a MPI equipment that can be used in the clinic.

### Photo-acoustic imaging (PAI)

The mechanism of photo-acoustic imaging relies on the excitation of a tissue with a nanosecond pulsed laser of 680–970 nm emission wavelength, 10 Hz frequency, and 10–100 mJ cm^−2^ power, resulting in a slight temperature increase and thermo-elastic expansion of the tissue, which further generates an acoustic wave that provides an image of tissue following detection and processing of the signal by an echograph of typical ultrasound frequency 5–50 MHz.^43–45^ Chromophores such as melanin and hemoglobin that are strong optical absorbers can act as intrinsic PAI contrast agents. However, in some cases they are not available, *e.g.* in some cancer cells, then requiring the use of other extrinsic PAI contrast agents such as IONP. IONP can behave as PAI contrast agents without the presence of another substance. In this case, the coupling mechanism between the incident laser light and IONP can involve surface plasmon waves.^46^ IONP can also be conjugated to a fluorophore such as Indocyanine Green,^21^ which enhance laser light absorption. An important number of applications of PAI has been described,^45^ such as the imaging of certain cancers (breast, prostate, bladder, melanoma, ovarian), lymph nodes possibly containing metastasis, circulating tumor cells, as well as neonatal brain, gastrointestinal, thyroid, or intra-operative imaging. PAI displays a number of advantages such as: (i) an operating mechanism that does not use radiation, (ii) fast imaging acquisition, *i.e.* typically less than 1 minute,^47^ (iii) a relatively good resolution, *e.g.* ∼0.5 mm was reached for breast imaging,^48^ which can be increased by increasing the ultrasound frequency, (iv) the relatively large area that can be covered with one scan, *e.g.* a surface of 10–50 cm^2^ of a breast tumor can be imaged,^48^ (v) the possibility to obtain functional information derived from a variation in hemoglobin or melanin concentration, (vi) the ability to combine PAI with other imaging techniques such as MRI or CT. However, this technique also suffers from a relatively low penetration depth (typically of the order of a few cm), which is due to the use of laser light and to the ultrasound frequency when it is too high, and to a too small number of PAI equipment that can be used in the clinic. As an example, the Twente Photoacoustic Mammoscope (PAM) was built at the University of Twente (Enschede, the Netherlands) to image breast tumor cells in the clinic, but this type of equipment remains a prototype.^49^ It needs to be more widely developed and made available in different hospitals to spread out the use of the PAI imaging technique.

### Scanner, computing tomography (CT)

X-ray scanners, also designated as computing tomography (CT), expose patients to an X-ray beam, resulting in a non-uniform absorption of X-rays by the patients, *i.e.* the absorption is different parts for various parts of the organism depending on their consistency (*Z*-number). Then the transmitted (non-absorbed) X-rays are detected, providing an image of these different parts with various contrasts. In fact, the interaction between X-rays and the organism is due to the photo-electric effect, whose strength is mainly proportional to *Z*^3^ (*Z*: atomic number), and Compton scattering, which is enhanced at high electron and mass density.^50^ The unit of attenuation measurement obtained in CT is Hounsfield unit (HU), leading to low CT number (HU value) for weakly absorbing material (−1000 HU for air and 0 HU for water), to intermediate HU values for slightly absorbing material (40 to 80 HU for tissue), and to larger HU values for stronger absorbing material (400 to 1000 HU for bone).^51^ Substances with high HU values (strongly attenuating X-ray) appear white or light gray, while those with low HU values (weakly attenuating X-ray) appear dark gray or black. The photoelectric and Compton effects suggest that material with high *Z* and large mass density would be the best contrast agent. Although the *Z* value of iron, *i.e.* 26, and the density of maghemite comprised in most IONP for medical application, *i.e.* 5 g cm^−3^, are not the largest values that can be reached with nanomaterials, and are larger than those of water (*Z* = 1 for hydrogen, and *Z* = 8 for oxygen, *r* = 1 g cm^−3^ for water) that constitutes the majority of organic living material, they seem sufficient to provide. They seem are sufficient to provide a contrast with CT. Indeed, IONP were shown to act as CT contrast agent, leading to CT number that increases with increasing IONP concentration, *i.e.* from 27 HU at 2.5 mg mL^−1^ of IONP to 113 HU at 25 mg mL^−1^ of IONP.^52^ However, compared with iodine, IONP were shown to absorb ∼5–6 times less X-ray.^52^ The advantages of CT lie in: (i) the acquisition of large scans (typically 10–20 cm) quickly (under 5 seconds) with image reconstruction within less than one minute, (ii) its non-invasiveness, (iii) high contrast resolution, (iv) no depth penetration limit, (v) a relatively low cost, (vi) quantitative information on contrast agents can be obtained, (vii) its wide availability in hospitals. Disadvantages of CT come from: (i) its low sensitivity compared with other imaging techniques, *i.e.* CT detection limit is ∼10^−3^ M,^51^ compared with 10^−5^ M for Gd chelates in MRI and 10^−9^ M for nuclear techniques about,^51^ (ii) the exposure of patient to relatively high dose of ionizing radiation, (iii) limited soft tissue resolution.

### Positron emission tomography (PET)/single photon emission computed tomography (SPECT)

The mechanism of PET and SPECT relies on the use of a radio-tracer, which targets a specific part of the organism such as a tumor, then emits a signal made of positrons (PET) or gamma rays (SPECT) that is measured, hence enabling the detection of this part of the organism. PET and SPECT are functional imaging methods mimicking a substance of interest such as glucose for ^18^F-FDG, which gets fixed on a specific tissue, *e.g.* a tumor in case of ^18^F-FDG that consumes more glucose than a healthy tissue.^53^ Using IONP, it was possible to bring several improvement to the standard PET/SPECT imaging method by: (i) binding different radio-tracers to IONP [18F]fluorodeoxyglucose (FDG), copper-61/64, gallium-66/68, zirconium-89, and iodine-124 for PET,^54^ and 99mTc, 125I, 111I, 125I and 131I for SPECT,^54–56^ that increase radio-tracer lifetime and targeting efficacy,^55^ (ii) enabling simultaneous anatomical and functional imaging by combining PET/SPEC with MRI, taking advantage of the MRI contrasting ability of IONP, (iii) enlarging the SPECT/PET imaging capacity to a therapeutic activity through the use of a theranostic IONP probe that can trigger drugs delivery, immunotherapy, hyperthermia, or photodynamic therapy.^19,57^ In addition, PET/SPECT lead to high detection sensitivity, *e.g.* PET was shown to be 200 times more sensitive than MRI using nanoparticles, the possibility to image the whole organism without limitation in depth of penetration, and the acquisition of quantitative information since in optimal operating conditions PET/SPECT signal should be proportional to the number/concentration of radiotracer in the targeted region.^54^ For these reasons, the development of PET/SPECT can be foreseen in various medical fields such as disease detection or the assessment of the efficacy of a medical treatment.

### Ultrasound imaging/sonography

It was reported that IONP could be used in ultrasound (sonography) imaging. On the one hand, IONP could be inserted within a material that is already an ultrasound contrast agent such as a micro-bubble or liposomes, then enabling a combination between USI and another imaging modality such as MRI that is made possible by the presence of IONP.^58^ On the other hand, although a direct detection of IONP by ultrasound was not reported to the author knowledge, IONP presence could be detected indirectly by measuring tissue resistance resulting from IONP motion/vibration under the application of a high intensity pulsed magnetic field through a technique cold magneto-motive ultrasound whose efficacy essentially depends on IONP susceptibility and could be combined with ultrasound imaging to provide both anatomical tissue information and estimate the quantity of IONP or photo-acoustic imaging to improve the resolution of PAI imaging. Magneto-motive approaches could be used to carry out dynamic imaging, *i.e.* imaging of moving biological systems, such as circulating tumor cells, metastases diffusing in/out of lymph nodes or stem cells, to determine viscoelastic property of soft tissues, and could easily be combined with other imaging modalities.^59^

### Optical imaging methods

Optical imaging can be carried out with IONP-fluorophore complexes, essentially using NIR fluorophores such as organic fluorochromes, *e.g.* cyanine dyes such as Cy5.5. Such complexes are either made of IONP surrounded by various coatings such as silica shell, spacer, lipid bilayer, polymer, which contain or are labeled with fluorophores or of IONP directly coated with a fluorescent semiconducting material. Such methods present the advantages of being relatively easy to implement, of yielding under optimized conditions a high spatial/temporal resolution, and of being complementary to other imaging techniques such as MRI. In order for this method to be efficient, absorption by the organism/tissue/water/hemoglobin/melanin/proteins that occurs between 200 and 650 nm shall be avoided by choosing fluorescent probes with absorption/emission within the range of 650–1450 nm. Furthermore, fluorophores should not be prone to photo-bleaching, not be quenched by iron oxide, be stable in biological media, *i.e.* have a long life-time in such environment, and display well-separated emission and absorption spectra to be to distinguishable them. The use of this method is facilitated by the existence of numerous systems that can excite/detect fluorescence in different conditions, *i.e.* in real time, *in vitro*, *in vivo*, such as: optical scanners, *e.g.* fluorescence mediated tomography, fluorescence reflectance tomography, optical coherence tomography, a large number of fluorescence microscopies, flow cytometry, spectrophotometry, intra-vital microscopy, intravascular non-invasive near-infrared (NIRF) imaging, clinical endoscopy, and equipment for fluorescence detection during surgery. Such method can be used for monitoring magnetofection efficacy,^60^ for multi-modal imaging with MPI, MRI and PAI,^61,62^ for detecting various biological entities such as tumors,^16,56^ apoptotic cells,^7^ and sentinel lymph nodes,^4^ and for delineating infiltrating tumors such as glioblastoma.^63^

### Multimodal imaging

Since IONP can be used with various imaging methods, they could be considered for multimodal imaging. The latter presents the advantage of enabling the combination of information coming from different imaging methods.^64^ For example, in PET/MRI, MRI is used for anatomical imaging while PET offers molecular information.

## Conclusion/perspectives

From the author's point of view, the challenges ahead lie in:

• Improving both IONP (better targeting, size control, fabrication process) and imaging methods (better resolution, miniaturization, lower cost, more limited use of hospital infrastructure);

• Yielding a sufficiently large percentage of IONP in the organ of interest so that IONP can act as contrast agents;

• Developing a single imaging method that brings together the maximum benefit of the different imaging techniques with the minimum of their drawbacks;

• Building a device/platform that gathers all the different imaging techniques in one unit;

• Identifying the specific medical need upstream and then providing the imaging method best suited to this need, an approach that seems appropriate when medical needs remain globally unchanged, which may be the case within a limited time period in specialized hospital units.

• IONP present the advantage of being compatible and even improving a large series of different imaging techniques, making them appealing for multimodal imaging.^64^

## Abbreviations

BBB: Blood brain barrier
CA: Contrast agent
CT: Computing tomography
IONP: Iron oxide nanoparticles
MRI: Magnetic resonance imaging
MPI: Magnetic particle imaging
MPH/MHT: Magnetic particle hyperthermia
MPS: Mononuclear phagocytic system
MS: Multiple sclerosis
OI: Optical imaging
PAI: Photo-acoustic imaging
ROS: Radical oxygen species
US: Ultrasounds
USI: Ultrasound imaging (sonography)

## Conflicts of interest

EA has been working in the company Nanobacterie.

## Supplementary Material
